# Myricetin as a Potential Adjuvant in Chemotherapy: Studies on the Inhibition of Human Glutathione Transferase A1–1

**DOI:** 10.3390/biom12101364

**Published:** 2022-09-24

**Authors:** Mohammed Hamed Alqarni, Ahmed Ibrahim Foudah, Magdy Mohamed Muharram, Aftab Alam, Nikolaos E. Labrou

**Affiliations:** 1 Department of Pharmacognosy, College of Pharmacy, Prince Sattam Bin Abdulaziz University, P.O. Box 173, Alkharj 11942, Saudi Arabia; 2 Department of Microbiology, College of Science, Al-Azhar University, Nasr City, Cairo 11884, Egypt; 3 Laboratory of Enzyme Technology, Department of Biotechnology, School of Food, Biotechnology and Development, Agricultural University of Athens, 75 Iera Odos Street, GR-11855 Athens, Greece

**Keywords:** flavonoids, glutathione transferase, multi-drug resistance, enzyme inhibition

## Abstract

Glutathione transferases (GSTs) are a family of Phase II detoxification enzymes that are involved in the development of multi-drug resistance (MDR) phenomena toward chemotherapeutic agents. GST inhibitors are considered candidate compounds able to chemomodulate and reverse MDR. The natural flavonoid myricetin (MYR) has been shown to exhibit a wide range of pharmacological functions, including antitumor activity. In the present work, the interaction of MYR with human glutathione transferase A1–1 (hGSTA1–1) was investigated by kinetics inhibition analysis and molecular modeling studies. The results showed that MYR binds with high affinity to hGSTA1–1 (IC50 2.1 ± 0.2 μΜ). It functions as a non-competitive inhibitor towards the electrophile substrate 1-chloro−2,4-dinitrobenzene (CDNB) and as a competitive inhibitor towards glutathione (GSH). Chemical modification studies with the irreversible inhibitor phenethyl isothiocyanate (PEITC), in combination with in silico molecular docking studies allowed the prediction of the MYR binding site. MYR appears to bind at a distinct location, partially overlapping the GSH binding site (G-site). The results of the present study show that MYR is a potent inhibitor of hGSTA1–1 that can be further exploited towards the development of natural, safe, and effective GST-targeted cancer chemosensitizers.

## 1. Introduction

Glutathione transferases (GSTs) are multifunctional enzymes that have evolved from thioredoxins/glutaredoxins family [1,2,3,4]. They contribute to biotic/abiotic stress responses by eliminating reactive electrophile species [1,2,5,6,7,8]. The human GST gene family comprises sixteen genes that are grouped in six subfamilies: alpha (GSTA), mu (GSTM), omega (GSTO), pi (GSTP), theta (GSTT), and zeta (GSTZ) [9,10]. The human alpha class GSTs subfamily includes five isoenzymes (hGSTA1–1 to hGSTA5–5), whose genes are located on chromosome 6p12.1–6p12.2. The isoenzyme hGSTA1–1 appears to be involved in a wide range of functions [10,11,12,13,14,15,16]. It is expressed at high levels in liver, intestine, kidney, adrenal gland, and testis, and catalyzes the detoxification of a wide range of carcinogenic metabolites and several alkylating chemotherapeutic agents that are used in cancer therapy [10,11,12,13,14,15,16]. It also exhibits high peroxidase activity toward fatty acid and phosphatidyl hydroperoxides [10].

Intense research over the past two decades has established that GSTs are key enzymes that are directly connected to the therapeutic response to chemotherapy. High expression levels of GSTs are connected with an increased resistance of tumors to a variety of anticancer drugs [4,5,6,11]. They are considered druggable targets for managing MDR cells, because their inhibition can directly affect metabolic pathways and cell signaling [11,12]. Several pieces of evidence have shown that GSTs are involved in MDR phenomenon through both a catalytic (ability to metabolize and inactivate anticancer agents) as well as a noncatalytic (regulation of cell-signaling mechanisms) function [5,11,13,14,15]. It is well established that the catalytic and noncatalytic roles of hGSTA1–1, in combination with its overexpression in several types of cancer cells, contribute to the development of MDR [14,15,16]. For instance, it has been found that hGSTA1–1 promotes lung cancer cell invasion and adhesion and mediates the effect of nicotine on lung cancer cell metastasis in vitro [13]. The enzyme exerts its effect by promoting the epithelial-mesenchymal transition, a process that is strongly associated with lung cancer metastasis [13]. In another example, Zou et al., 2019 [14] demonstrated that hGSTA1–1 is involved in cisplatin resistance of common types of solid cancer and concluded that specific hGSTA1–1 inhibitors may act as general sensitizers of the common types of solid cancer cells to cisplatin cytotoxicity through the promotion of cell apoptosis. In addition, Liu et al., 2018 [15] showed that hGSTA1–1 suppressed tumor growth and induced cell apoptosis in A549 cell line, indicating that hGSTA1–1 contributes to the regulation of cell proliferation and apoptosis. More recently, Teslenko et al., 2022 [16] showed that the hGSTA1*B allele affects the metabolism of exemestane, an aromatase inhibitor, in human liver cytosols and can be therefore considered as an important biomarker for the therapeutic outcome and toxicity [16].

Polyphenols are natural compounds with variable structures that are classified into several subclasses including catechins, flavonoids (flavonols, flavanols, flavones, isoflavones), anthocyanins, chalcones, curcuminoids, and phenolic acids. Flavonoids are being increasingly studied for their diverse biological activities, including anticancer activity, and negligible side effects [17,18,19]. The idea of using polyphenols for cancer therapy or as potential adjuvants in chemotherapy is not new. Early studies, carried out in the late twentieth century, established the anti-cancer effects of different polyphenols. Since then, significant progress has been made on understanding their multifunctional roles [17,18,19,20].

**Figure 1:**
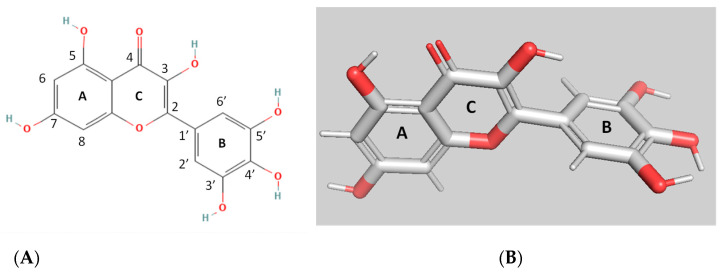
The 2D (**A**); and 3D (**B**) structures of myricetin (MYR).

The present work aims to investigate the interaction of myricetin (MYR) with hGSTP1–1. MYR (Figure 1) is a polyphenolic compound that belongs to the flavonoid class. It is composed of three aromatic rings, A, B, and C. The presence of 2,3-double bond in the C ring increases the planarity of the molecule; thus it adopts rigidity, holding A and C rings in a coplanar position.

MYR is one of the most common flavonoids in fruits, vegetables, herbs, tea, and red wine and displays a range of diverse bioactivities in different cell processes [21,22], such as apoptosis, glycolysis, cell cycle, energy balance, lipid level, and osteoclastogenesis [21,22,23,24]. In addition, MYR has been shown to affect cancer cells through diverse mechanisms. For example, it suppresses cancer cell invasion and metastasis, induces cell cycle arrest and apoptosis, and inhibits cell proliferation [25,26,27]. Furthermore, MYR has been shown to promote apoptosis by regulating Bcl−2 proteins, MAPK, and Wnt/*β*-catenin signaling, and by stimulating ROS-mediated stress, endoplasmic reticulum stress, and DNA damage [21,22,23,24]. MYR is also involved in modulating other cell pathways such as PI3K/Akt pathway, Nrf2 signaling, mTOR pathway, Ras/Raf pathway, and JAK/STAT pathway and Btk [28]. MYR has also been shown to regulate the expression of inflammatory factors, induce protective autophagy, induce cell cycle arrest, inhibit cell invasion, and inhibit migration [24,25,26]. Several studies have demonstrated the in vitro role in MYR in chemoresistance. For example, Zheng et al., 2017 [29] showed that myricetin induces apoptosis and enhances chemosensitivity in ovarian cancer cells. In another work, Huang et al., 2015 [30] demonstrated that myricetin inhibits proliferation of cisplatin-resistant cancer cells and could potentially be used to overcome cancer chemoresistance against platinum-based therapy.

Considering that MYR contributes to chemoresistance and taking into account the key role of hGSTA1–1 in MDR mechanism, the present work was undertaken in order to evaluate whether the activity of hGSTA1–1 can be manipulated by MYR. The results of the present study shine light on the role of MYR as potent inhibitor towards the MDR-involved hGSTA1–1. The outcome of the work provides new insights into the drug design effort towards MDR targets that can facilitate the rational development of GST-targeted chemosensitizers.

## 2. Materials and Methods

### 2.1. Materials

Reduced glutathione (GSH), 1-chloro−2,4-dinitrobenzene (CDNB), bovine serum albumin (BSA), MYR, and all other buffers, media, and salts were purchased from Sigma–Aldrich (St. Louis, MO, USA).

### 2.2. Heterologous Expression and Purification of Recombinant hGSTA1–1

Recombinant hGSTA1–1 was expressed in *E. coli* BL21 (DE3) cells and purified by affinity chromatography on immobilized GSH as described elsewhere [31,32,33]. Protein concentration was determined by the method of Bradford, using BSA as standard.

### 2.3. Assay of hGSTA1–1 Activity and Inhibition Analysis

The activity of hGSTA1–1 was assessed by measuring the conjugating activity of the 1-chloro−2,4-dinitrobenzene (CDNB)/GSH system as described previously [31,32,33]. For the determination of the concentration of inhibitor at which 50% inhibition of enzyme activity was obtained (IC50), the reaction mixture contained different concentrations of MYR (0–2.3 μΜ). The IC50 values were determined by fitting the following equation to the concentration-response data:

%Inhibition = 100/[1 + (IC50/[I])]

where [I] is the concentration oy MYR. The IC50 values were determined using the program GraphPad Prism 5 (GraphPad Software, San Diego, CA, USA).

### 2.4. Kinetic Inhibition Studies

Measurement of the dependence of the initial rates of catalytic activity on CDNB concentration was carried out in 0.1 M potassium phosphate, pH 6.5, (37 °C), using 0.02–1 mM CDNB in the presence of 2.5 mM GSH in the presence or in the absence of MYR (0–2 μΜ). Measurement of the dependence of the initial rates of catalytic activity on GSH concentration was achieved using 0.035–2.5 mM GSH and 1.5 mM CDNB, in the presence or in the absence of MYR (0–8 μΜ). Measurements of initial rates were carried out in triplicate. The kinetic data were analyzed by nonlinear regression analysis using the computer program Graph PadPrism 5 (GraphPad Software, San Diego, CA, USA).

### 2.5. Irreversible Inactivation of hGSTA1–1 by Phenethyl Isothiocyanate (PEITC)

Irreversible inactivation of hGSTA1–1 by PEITC [34] was performed at 37 °C in an incubation mixture (1 mL) containing potassium phosphate buffer (0.1 Μ, pH = 7.5), PEITC (0.5 mM) and hGSTP1–1 (approximately 0.1–0.2 U). The course of inactivation was followed by periodically removing samples for assaying the hGSTA1–1 activity. Initial rates of enzyme inactivation were determined from plots of log(% remaining activity) versus time (min) [32,33,35,36]. Irreversible inactivation of hGSTP1–1 by PEITC was also carried out in the presence of S-nitrobenzyl-glutathione (1 mM) or MYR (10–50 μΜ) as described above.

### 2.6. Molecular Modeling

Docking calculations for the MYR and hGSTA1–1 interaction were carried out by AutoDock Vina [37], with default parameters. The structure of hGSTA1–1 with PDB code 1k3y was used in docking studies [38]. For inspection of models and crystal structures the programs PyMOL (http://www.pymol.org/, accessed on 9 August 2022) [39] and UCSF Chimera [40] were used.

### 2.7. Statistical Analysis

The presented data were obtained from three independent measurements. Graph PadPrism 5 (GraphPad Software, San Diego, CA, USA) were used for data analysis and evaluation.

## 3. Results and Discussion

### 3.1. Kinetics Inhibition Analysis of the Interaction of MYR with hGSTA1–1

**Figure 2:**
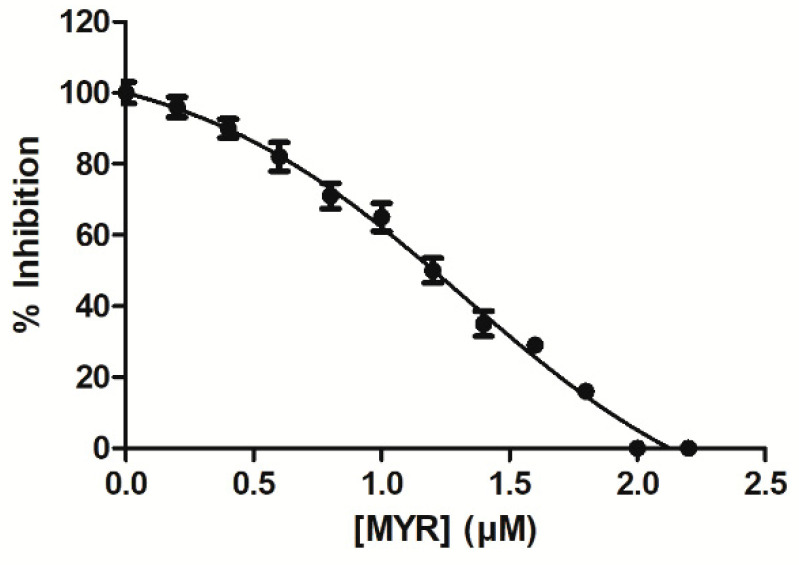
Concentration-response curve for the determination of IC50 value for the inhibition of hGSTA1–1 by MYR. The graph was produced using GraphPad Prism 5. The IC50 value corresponds to the concentration of inhibitor that gives 50% inhibition in the standard assay system with CDNB and GSH.

The interaction of MYR with hGSTA1–1 was studied by employing inhibition studies. The GST substrate system GSH/CDNB was selected for measuring the effect of MYR on enzyme activity. hGSTA1–1 is highly efficient in catalyzing the conjugation of GSH to CDNB, allowing the determination of kinetic constants with high accuracy and reproducibility [39,41]. Figure 2 shows the dose response curve for the inhibition of the enzyme by MYR. The IC50 value for MYR was determined 2.1 ± 0.2 μΜ, suggesting that MYR is a strong inhibitor towards hGSTA1–1. The low IC50 determined for MYR compares favorably to those that have been reported in the literature [31,35,42,43,44,45,46]. For example, the IC50 values for colchicine [43], for the synthetic 2-(pyrrolesulfonylmethyl)-*n*-arylimines, and benzophenones and their carbonyl *n*-analogues were in the range 22–71 μΜ [45]. However, ethacrynic acid inhibits hGSTA1–1 with Ki (μM) of 4.6–6.0 [47]. Ethacrynic acid was shown to enhance the cytotoxicity of anticancer drugs chlorambucil in several cancer cell lines and melphalan in xenograft models in SCID mice [48]. Furthermore, a significant chemosensitizing activity of ethacrynic acid has also been reported in patients [49]. However, important side effects have been reported for ethacrynic acid, making it less suitable for clinical applications [50].

Kinetic inhibition studies were carried out aiming to obtain kinetics data for shining light on the type of inhibition as well as a roughly prediction of the location of its binding site on hGSTA1–1. The enzyme possesses three discrete ligand binding sites. The first is the conserved GSH binding site, “G-site”, which is located at the enzyme’s *n*-terminal domain I. The second, “H-site”, is found at the C-terminal domain II, which binds the hydrophobic electrophilic acceptor substrates [4,34,36,38]. This pocket creates a hydrophobic environment and is mostly formed by non-conserved residues. Different substrate specificities depend on the shape and physicochemical properties of the H-site, which is mainly determined by residues in the β1–α1 loop, the C-terminal part of the helix α4 and the C-terminus [10,34,38,46,51,52]. hGSTA1–1 contains a further non-catalytic ligandin binding site “L-site”, which is involved in binding of non-substrate metabolites. In GSTs, the presence or location of the L-site is not a conserved feature, it can be situated at the dimer interface or at a region that overlaps the H- and the G-site [4,34,36].

**Figure 3:**
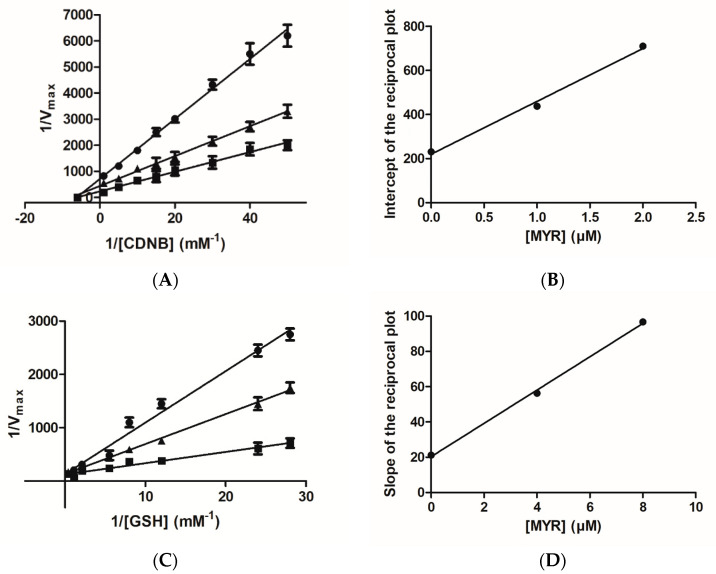
Lineweaver–Burk plots for the inhibition of hGSTA1–1 by MYR. (**A**) Inhibition of hGSTA1–1 by MYR (0 μM (■), 1 μM (▲), 2 μM (●)) using the concentration of GSH constant and the concentration of CDNB varied (0.02–1 mM); (**B**) The dependence of the intercepts of the Lineweaver–Burk plots on MYR concentration. (**C**) Inhibition of hGSTA1–1 by MYR (0 μM (■), 4 μM(▲), 8 μM(●)) using the concentration of CDNB constant and the concentration of GSH varied (0.035–2.5 mM). (**D**) The dependence of the slopes of the Lineweaver–Burk plots on MYR concentration.

The inhibition of hGSTA1–1 by MYR in the form of the Lineweaver–Burk plots is illustrated in Figure 3. MYR appears to behave as a non-competitive inhibitor (K_i_ = 1.1 ± 0.1 μΜ) (Figure 3A) against CDNB, as the lines of the Lineweaver–Burk plot intersect the CDNB concentration axes. The dependence of the slopes of the Lineweaver–Burk plots on MYR concentration was linear (Figure 3B), suggesting a fully non-competitive inhibition pattern. This type of inhibition arises when the enzyme binds with inhibitor (I) and the substrate (S), allowing the formation of ES, EI, and EIS complexes. The observed non-competitive inhibition pattern suggests that MYR binds at a discrete region, presumably localized out of the H-site, and the formation of E-CDNB-MYR complex is kinetically feasible. When GSH was used as a variable substrate, MYR behaved as a competitive inhibitor (K_i_ = 2.1 ± 0.2 μΜ), as the lines of the Lineweaver–Burk plot intersect the velocity axes (Figure 3C). The dependence of the slopes of the Lineweaver–Burk plots on MYR concentration was again linear (Figure 3D), suggesting a fully competitive inhibition pattern. The fully competitive inhibition happens when the enzyme (E) binds with the substrate (S) and inhibitor (I) separately, allowing the formation of ES and EI complexes. This type of inhibition suggests that MYR competes with the GSH for binding to the G-site of the enzyme. This inhibition pattern is different to that observed for the flavonoid fisetin, a structural analogue of MYR [31]. The observed competition of MYR with GSH for the same binding site (e.g., G-site), despite their structural dissimilarity, suggests that the overall 3D physicochemical features of MYR (e.g., high hydrophilicity) is the driving force for its preference for binding to the G-site instead of the H-site. The differences in the amino acid composition and physicochemical features (e.g., size, shape, and hydrophobicity) between the G- and H-site seem to provide the structural determinants that contribute to the molecular recognition and binding preferences of MYR. The GSTs that belong to the alpha class, have a smaller H-site than the GSTs of mu class [10]. Furthermore, the H-site in the alpha class enzymes is very hydrophobic (e.g., formed by Leu107, Leu108, Val111, Met208, Leu213, Phe222, Phe10, and Phe220), compared to other isoenzymes (e.g., hGSTP1–1), which exhibit a mixed function feature, composed of both hydrophobic and hydrophilic residues [10,34,36,38,51,52].

### 3.2. Irreversible Inactivation of hGSTA1–1 by Phenethyl Isothiocyanate (PEITC)

**Figure 4:**
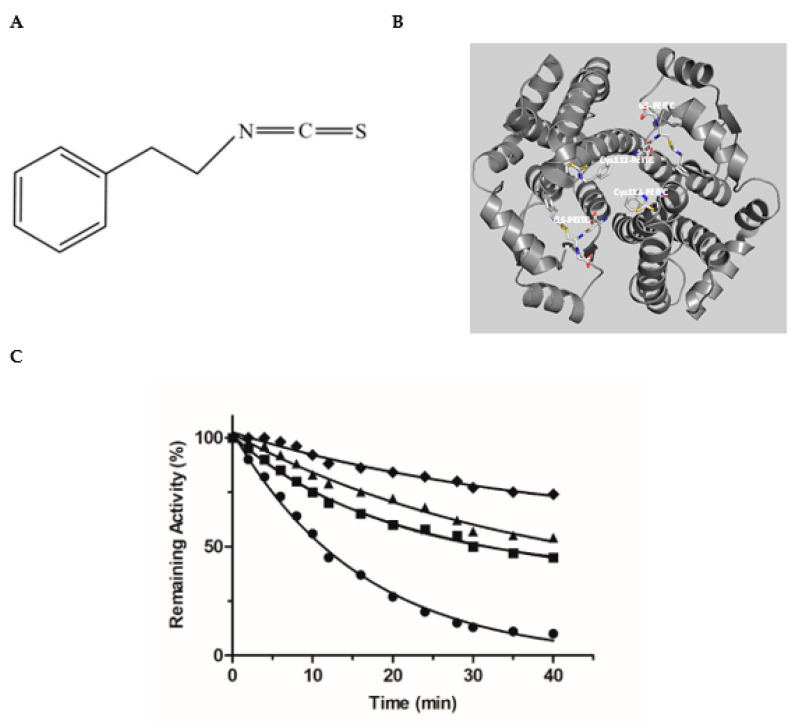
(**A**) The structure of PEITC. (**B**) The interaction of the Cys112-PEITC and the GS-PEITC (the adduct formed between GSH and PEITC) with hGSTA1–1 (PDB entry 5JCU). The Cys112-PEITC and GS-PEITC adducts are illustrated as sticks and the protein structure as a ribbon diagram. Nitrogen atoms are shown in blue, oxygen in red, and sulfur in yellow. (**C**) Time course of the hGSTA1–1 inactivation by PEITC: 0.5 mM PEITC (●); 0.5 mM PEITC in the presence of S-nitrobenzyl-GSH (1 mM, ■); 0.5 mM PEITC in the presence of MYR (10 μΜ, ▲); 0.5 mM PEITC in the presence of MYR (20 μΜ, ♦).

To further validate the inhibition pattern obtained by kinetics inhibition studies chemical modification and inactivation experiments were employed. Phenethyl isothiocyanate (PEITC) (Figure 4A) is an irreversible (covalent) inhibitor towards hGSTA1–1 [34]. PEITC is an isothiocyanate with a phenethyl group attached to the nitrogen. Naturally it is found in cruciferous vegetables and exerts an apoptotic function against tumor cells. Based on crystallographic (PDB entry 5JCU) and protein chemistry evidence, it has been reported that PEITC specifically reacts with the H-site residue Cys112 of hGSTA1–1 [28]. In particular, the sulfhydryl group of Cys112 acts as a nucleophile, attacking the isothiocynate central C atom of PEITC, leading to the formation of a covalent adduct with hGSTA1–1 (Figure 4B). Cys112 is a highly reactive residue in hGSTA1–1 and has been reported to react irreversibly with several electrophile compounds such as acetaminophen [53] or the chemotherapeutic drug chlorambucil [51].

Incubation of hGSTA1–1 with PEITC, leads to a time-dependent loss in enzyme activity, as expected for an irreversible inhibitor (Figure 4C). Incubation of hGSTA1–1 with PEITC in the presence of the inhibitors MYR or S-nitrobenzyl-GSH, diminishes the rate of enzyme inactivation (k_obs_) (Table 1). The presence of MYR or S-nitrobenzyl-GSH in the incubation mixture appears to protect the enzyme from inactivation by PEITC, suggesting that these compounds compete with PEITC for binding to the same site.

As we have already mentioned, PEITC specifically reacts with Cys112 of hGSTA1–1 [34] (Figure 4B). From the analysis of the enzyme crystal structure, it is evident that Cys112 is situated on the loop connecting helices H4 and H5, close to the G-site. Its side chain -SH is accessible for covalent modification by PEITC and projects into the large, solvent-filled cleft which is widely reported in the literature to be the binding site of non-substrate ligands [51,53]. The competition observed between PEITC and MYR (Figure 4C), supports the outcome of the kinetics inhibition studies and points to the conclusion that the binding-site of MYR is located at or close the G-site.

### 3.3. Study of the Interaction of hGSTA1–1 and MYR by In Silico Molecular Docking

**Figure 5:**
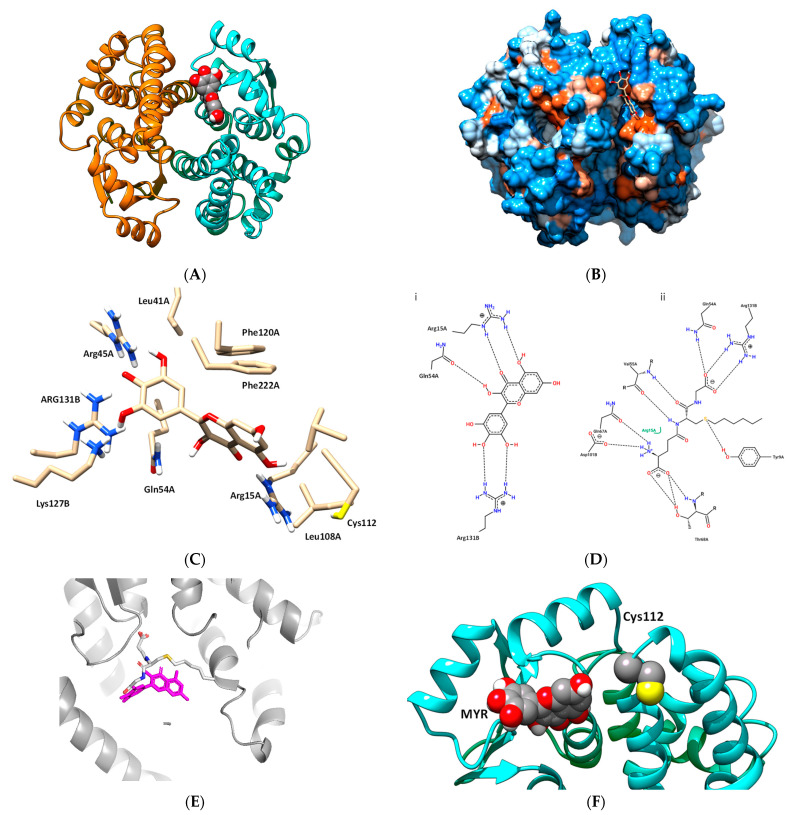
(**A**) The predicted interaction of MYR with hGSTA1–1. MYR bound to the hGSTA1–1 is shown in a ball representation and colored according to the atom type (gray: carbon; blue: nitrogen, red: oxygen). (**B**) The hydrophobicity surface of hGSTA1–1 is depicted and MYR is shown in a stick representation and colored according to the atom type. (**C**) A close-up view of the interactions of MYR with hGSTA1–1. Important side chains that contribute to interaction are shown in a stick representation and labeled. (**D**) Hydrogen bond interaction pattern of MYR (i), and S-hexyl-GSH (ii), upon binding to hGSTA1–1. The figure was created by PoseView [54]. (**E**) Superposition of the MYR and S-hexyl-GSH binding modes. Both ligands are shown in a stick representation. MYR is colored magenta and S-hexyl-GSH is colored according to the atom type. (**F**) The close proximity of MYR, bound to hGSTA1–1, with Cys112.

Prediction of the binding more of MYR to the hGSTA1–1 was carried out using in silico molecular docking performed by AutoVina [37]. The work aimed at predicting the possible interactions between the two molecules. The most favorable binding mode (docking score−8.6) of MYR with hGSTA1–1, is depicted in Figure 5A,B. MYR binds presumably to a specific pocket that is located at the solvent channel. Specific amino acid residues that contribute to the formation of the binding-site include a range of polar (e.g., Lys126, Arg130, Arg44, Arg14, and Gln53) and non-polar (Leu40, Phe221, Phe119, Val110, and Leu107) residues, suggesting that the interaction of MYR with hGSTA1–1 is governed by both polar and non-polar interactions (Figure 5C). Important residues, shown in Figure 5D(i), that contribute to a hydrogen bond formation with MYR include Arg15, Gln54, and Arg131. These residues are conserved in all GSTs and play crucial roles in G-site formation and GSH recognition, as shown in Figure 5D(ii) [34,36,51]. The B ring of MYR arrears to occupy the region that the Gly-moiety of GSH binds to the enzyme as illustrated in Figure 5C–E. This interaction is governed by Arg131 which appears to be crucial for positioning the B ring of MYR to the G-site. Therefore, the inhibition pattern observed by kinetics inhibition studies is in agreement with the in silico molecular docking results. Hydrophobic and van der Waals interaction also contribute to MYR binding, and involve Leu40, Phe221, Phe119, Val110, and Leu107, that form a hydrophobic wall that stabilize the two-ring (rings A and C, see Figure 1) system. Notably, the C-terminal residue Phe222 forms a π-π interaction with ring A of MYR.

The predicted binding of MYR appears to be in agreement with the results obtained by chemical modification studies, and confirmed the competition observed between PEITC and MYR or S-hexyl-GSH (Figure 4C). Figure 5F illustrates the close proximity of MYR and Cys112. It is conceivable to be proposed that the presence of MYR restricts the access of PEITC for chemical modification of Cys112, causing a substantial reduction of the observed rate of inactivation (Table 1).

## 4. Conclusions

This study expands the role and function of MYR, a widespread flavonoid, and establishes its role as a potent inhibitor towards hGSTA1–1. MYR is able to bind specifically to hGSTA1–1 at a distinct location at the solvent channel partially occupying the entrance of the G-site. The results of the present work suggest that MYR holds the potential for application as adjuvant in combination with drugs known to be substrates of hGSTA1–1 enzyme. The combination of MYR and a drug can affect cancer cells, overexpressing hGSTA1–1, in two discrete ways: firstly by preventing the drug’s metabolism and detoxification through the direct inhibition of hGSTA1–1 catalytic activity, and secondly, MYR can act as an anticancer compound. The outcome of the study can accelerate the development of GST-targeted cancer chemosensitizers. Future work involving in vivo and clinical studies will further evaluate and assess the efficiency, safety, and clinical use of MYR.

## Figures and Tables

**Table 1:** Observed rates and half-time of hGSTA1–1 inactivation by PEITC in the presence of the competing ligands S-nitrobenzyl-GSH and MYR.

Competing Ligand	k_obs_ (min ^−1^)	Half-Life (min)
-	0.062 ± 0.004	11.23
S-nitrobenzyl-GSH (1 mM)	0.049 ± 0.004	14.05
MYR (10 μΜ)	0.027 ± 0.006	25.84
ΜΥΡ (20 μΜ)	0.02589 ± 0.009	27.85

## Data Availability

Data are contained within the article.
